# SARS‐CoV‐2 booster immunization: stimulating de novo B‐cell response and antibody generation

**DOI:** 10.1002/mco2.465

**Published:** 2023-12-19

**Authors:** Qiaonan Hong, Ziyi Bai, Dong Tian

**Affiliations:** ^1^ Department of Thoracic Surgery West China Hospital, Sichuan University Chengdu China; ^2^ Lung Transplant Research Laboratory Institute of Thoracic Oncology, West China Hospital, Sichuan University Chengdu China

1

Alsoussi et al. recently reported in *Nature* ^1^ that developing vaccines derived from new variants can respond to the emergence of highly contagious variants. Boosting with the SARS‐CoV‐2 Omicron variant vaccine induces a de novo B‐cell response, which predominantly involves recruiting pre‐existing memory B cells. The research assessed the reaction of antigen‐specific B cells in humans to a booster immunization based on SARS‐CoV‐2 mRNA, which can further mature B cells and induce germinal center reaction. Additionally, the research showed that immunization using a monovalent B.1.1.529 BA.1‐matched vaccine led to a rare de novo B‐cell response, highlighting a novel epitope in the spike (S) protein of the B.1.1.529 BA.1 variant. This finding suggests the activation of naive B cells.

SARS‐CoV‐2 has a profound impact on people worldwide. Over time, the virus has demonstrated its ability to adapt continuously in response to the global population's immunity.^2^, ^3^ As a result, the primary vaccinations are no longer enough to meet people's needs, leading to a growing focus on booster immunization strategies. However, it has not been checked in humans whether re‐exposure to the primary strain or the variant of interest induces a solid germinal center (GC) reaction, which is crucial for improving high affinity and lasting antibodies.

First, Alsoussi et al. enrolled 46 healthy adults with no prior history of SARS‐CoV‐2 infection for their study. They had the third booster immunization of mRNA‐1273 or mRNA‐1273.213, following the completion of two doses of the primary mRNA vaccines (BNT162b2 or mRNA‐1273, both encoding complete sequence of SARS‐CoV‐2 S protein, based on the WA1/2020). In both cohorts, S‐specific immunoglobulin G (IgG) plasmablasts (PBs) were evaluated using an enzyme‐linked immune absorbent spot assay after 1 week. However, S‐specific IgA PBs in the mRNA‐1273.213 cohort were lower. The plasma antibody binding level of all strains increased within 4 weeks after immunization and decreased at 17 weeks. A strong S‐specific GC reaction could be seen in the subjects’ draining axillary lymph nodes, and persisted for at least 8 weeks after vaccination. Bone marrow plasma cells (BMPCs) producing IgG were detected in all participants who consented to collect bone marrow, and BMPCs were consistent with the continuous plasma antibody titer. Subsequently, to characterize the breadth of the boosted memory B cells (MBCs) pool, their binding of participant monoclonal antibodies (mAbs) to the S proteins of WA1/2020, B.1.351, B.1.617.2, and BA.1 strains were assessed using enzyme‐linked immunosorbent assay, and results showed that more than 90% of the mAbs recognized the S proteins of the WA1/2020, B.1.351, and B.1.617.2 strains, while approximately 60% identified the S protein of the BA.1 strain (Figure 1).

**FIGURE 1:**
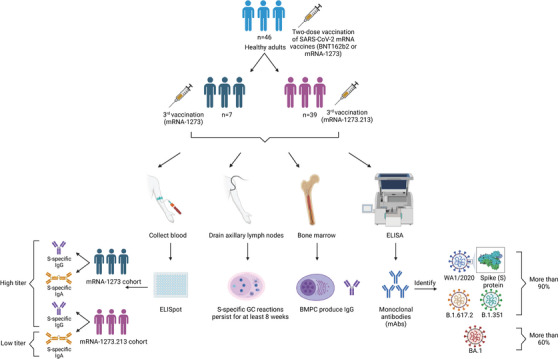
Participants were vaccinated with two‐dose of SARS‐CoV‐2 mRNA vaccines (BNT162b2 or mRNA‐1273) and then were injected with the third dose of mRNA‐1273 and mRNA‐1273.213 vaccines. Enzyme‐linked immune absorbent spot (ELISpot) showed different S‐specific immunoglobulin G (IgG) titer. Analysis axillary lymph nodes found that S‐specific germinal center (GC) reaction could last for at least 8 weeks. Bone marrow detect bone marrow plasma cell (BMPC) that produce IgG. Enzyme‐linked immunosorbent assay (ELISA) showed the binding ability of monoclonal antibodies (mAbs) to different S proteins.

After the booster immunization, scRNA‐seq analysis revealed that most identified S‐specific PB clones shared clonal relationships with MBCs, GC B cells, and/or plasma cells induced by initial vaccination. The PB response following the second dose and the booster injection within the primary vaccination revealed the presence of multiple S‐specific B‐cell clones. Compared to the clones involved in the primary response, the clones involved in the boosted PB response showed a notably higher frequency of somatic hypermutation (SHM), indicating their recall from affinity‐mature MBCs. Seventeen weeks after the booster vaccination, the SHM frequency of S‐specific MBCs significantly increased, surpassing that of clone‐related MBCs isolated 6 months after the primary vaccination, indicative of consistent GC‐driven maturation rounds. A similar trend was observed in paired S‐specific BMPC clones, both 6 and 9 months after the post‐primary immunization and 6 months after the booster injection. These results indicated that mRNA‐1273 and mRNA‐1273.213 encoding by B.1.351 and B.1.617.2, respectively, triggered strong GC reaction and MBC and BMPC responses maturation. However, no antibodies specifically targeting the variants encoded by the mRNA‐1273.213 vaccine were isolated, and there was no cross‐reaction with the original WA1/2020 S protein. Thus, the B‐cell response boosted with the mRNA‐1273.213 vaccine was primarily influenced by the primary vaccination series of mRNA‐1273 encoding the original S protein.

To assess whether a booster dose with an antigenically distinct formulation could elicit a detectable response to a new epitope, participants were administered a third dose of mRNA‐1273.529 following the completion of two doses of the mRNA vaccine targeting the ancestral strains. All participants had a strong circulating IgG‐producing PB response to the original WA1/2020 strain S protein and the vaccine‐encoded BA.1 S protein, with an MBC pool similar in breadth to the mAb from the mRNA‐1273 and mRNA‐1273.213 cohorts. The ability of the mAbs to neutralize a panel of authentic infectious SARS‐CoV‐2 variants was also evaluated, and it was found that the infection was reduced by at least 90% at 5 μg/mL. It is important to note that most isolated mAbs from BA.1‐specific MBCs, which do not bind to WA1/2020, still exhibited cross‐reactivity with the WA1/2020 S protein. This indicates that the S protein‐dependent sorting method may not be optimal for assessing the binding specificity of variants, as it fails to differentiate the binding specificities of variant proteins accurately. They tested the neutralizing ability of six mAbs, which only combined BA.1 with the SARS‐CoV‐2 variant, and found that they exhibited significantly reduced SHM levels and could not neutralize the primary strains. Subsequently, six BA.1‐specific mAb‐targeted amino acid residues were identified, and it is worth noting that many mutations were found to be the reversion of SARS‐CoV‐2 primary strains.

In summary, the research of Alsoussi et al. provide crucial theoretical support for popularizing booster immunization in the future, including the effectiveness and breadth of human serum antibody response improved after booster immunization. Booster immunization has several advantages. First, the B‐cell clones induced by booster immunization exhibit a higher mutation rate than identical clones detected 1 week after completing the initial vaccination series. Second, it has been observed that the MBCs generated during the initial vaccination series play a dominant role in the recall response elicited by booster immunization. Additionally, after boosting immunization, a new GC reaction can be stimulated. The frequency of BMPCs of IgG secreted by the original SARS‐CoV‐2 S protein is significantly higher, which compared to observed 7 months after mild SARS‐CoV‐2 infection or 6 months after the primary mRNA vaccination series. This highlights the crucial role of repeated antigens exposure in increasing the frequency of antigen‐specific BMPC.

MBCs produced by primary vaccination can form competitive clones that are more specific than variants, thus affecting the immunity of booster immunization. The research of Alsoussi et al. emphasizes that the study of de novo B cells is significant to deal with the variants that have not yet appeared. Several investigations have focused on using different vaccination strategies to amplify mutation‐specific B‐cell clones. For instance, Sokal et al. showed that compared with other variants, Omicron's specific mutation resulted in more diverse receptor binding domain (RBD)‐specific MBCs. A small number of seriously infected COVID‐19 donors vaccinated with the mRNA vaccine could produce effective neutralizing antibodies, but the neutralizing ability from other variants needs to be further evaluated.^4^ However, how to solve the influence of pre‐MBCs on booster immunization needs further research. To sum up, timely booster immunization is beneficial for inducing de novo B‐cell response, to cope with the rapid mutations of SARS‐CoV‐2.

## AUTHOR CONTRIBUTIONS

D.T. conceived the idea and planned the study. Q.H. and Z.B. drafted the manuscript. D.T. revised the manuscript. All authors read and approved the final manuscript.

## CONFLICT OF INTEREST STATEMENT

The authors declare they have no conflicts of interest.

## ETHICS STATEMENT

Not applicable.

## Data Availability

Not applicable.
